# The effect of mother’s educational status on early initiation of breastfeeding: further analysis of three consecutive Nepal Demographic and Health Surveys

**DOI:** 10.1186/s12889-015-2405-y

**Published:** 2015-10-19

**Authors:** Pawan Acharya, Vishnu Khanal

**Affiliations:** Nepal Development Society, Bharatpur, Nepal; School of Public Health, Curtin University, Perth, Australia

**Keywords:** Breastfeeding, Maternal educational status, Demographic and health survey, Nepal

## Abstract

**Background:**

The World Health Organization recommends initiating breastfeeding within the first hour of birth. This study is aimed at assessing the effect of the mother’s education on early initiation of breastfeeding.

**Methods:**

Data from the Nepal Demographic and Health Surveys (NDHS) 2001, 2006 and 2011 were used which included 12,845 last born children born within 5 years before the surveys. Early initiation of breastfeeding was defined as the initiation of breastfeeding within the first hour after birth. Hierarchical modelling was used to ascertain the association of maternal education and early initiation of breastfeeding, after controlling for other covariates in a multiple logistic regression.

**Results:**

Maternal education was associated with a higher likelihood of early initiation of breastfeeding in each survey. Pooled data analysis revealed higher odds of early initiation of breastfeeding among the mothers with primary education (adjusted odds ratio (OR) 1.24, 95 % confidence interval (CI): 1.09, 1.42) and secondary or higher education (OR: 1.63 95 % CI: 1.42, 1.88). In the most recent NDHS 2011 survey, odds of early initiation of breastfeeding was higher among mothers with primary education (OR: 1.52; 95 % CI: 1.21, 1.91) and mothers with secondary or higher education (OR: 2.20; 95 % CI: 1.76, 2.76) compared to mothers with no education. Similarly, the odds of early initiation of breastfeeding was higher among mothers with secondary and higher education in the 2006 data (OR: 1.66; 95 % CI: 1.30, 2.12) and in 2001 (OR = 1.30; 95 % CI: 1.00, 1.67).

**Conclusions:**

As the association between a mother’s educational status and her likelihood of early initiation of breastfeeding increases, long-term approaches to prioritising education for women and girls should be explored. In the short term, uneducated mothers should be targeted with breastfeeding promotion strategies such as counselling and peer education.

**Electronic supplementary material:**

The online version of this article (doi:10.1186/s12889-015-2405-y) contains supplementary material, which is available to authorized users.

## Background

Globally, about 60 % of child deaths are directly or indirectly linked to undernutrition. Two- thirds of child deaths are estimated to be attributed to improper feeding during the first year of life [1]. As the leading global health institution, the World Health Organization (WHO) has identified sub-optimal breastfeeding as a barrier to sustainable socioeconomic development and poverty reduction [1] and recommends initiating breastfeeding within the first hour after birth [2–4]. The first breast milk after delivery (colostrum) provides energy and immunity to newborn [5]. Delayed initiation of breastfeeding may lead to hypothermia [6]. A study has reported higher odds of neonatal mortality [pooled odds ratio (OR): 2.02, 95 % confidence interval (CI): 1.40, 2.93] among those who were breastfed after the first hour of birth compared to the children breastfed within the first hour after birth [7]. Similarly, a hospital based cross sectional study in Ghana reported an increased risk of infant mortality in the case of delayed initiation of breastfeeding. The study reported greater than a two-fold risk (OR: 2.4, 95 % CI: 1.69, 3.40) of mortality if the initiation was delayed for 1 day after birth [8]. In Nepal, the risk of infant mortality was found higher among those who were breastfed later than 24 h of birth (relative risk (RR): 1.41; 95 % CI: 1.08, 1.86) [9].

Nepal has achieved the target for the millennium development goal(MDG) target on the reduction of child mortality. However, neonatal mortality is still alarming at the rate of 33 per 1000 live births and was relatively stagnant in the past decade [10]. A cross-sectional study from central Nepal in 2005 reported that 72.7 % of recently delivered women initiated breastfeeding within the first hour of birth [11]. More recently, a community based prospective cohort study from central Nepal showed that about 67 % mothers initiated breastfeeding within first hour after delivery [12]. A further analysis of the NDHS 2011 reported that about 27 % of mothers provided prelacteal foods to their newborn babies [13]. That study found maternal education associated with the prelacteal feeding. Similarly, studies from abroad have found a positive association between maternal education status with the early initiation of breastfeeding [14, 15], exclusive breastfeeding practice [16–18] and the duration of breastfeeding [19]. However, early initiation of breastfeeding and the factors associated with early initiation remains understudied in Nepal.

Education status of the mother has been identified as an important social determinant of health for children. The role of maternal education on infant feeding behaviour is an interesting area to explore. Women’s education in Nepal has long been neglected and still remains much lower than men. In recent years, there has been an improvement in women’s education status. Therefore, the effect of maternal education might also impart effect on health and infant feeding behaviour. This study aims to investigate the association between early initiation of breastfeeding and mother’s educational status using data from the Nepal Demographic and Health Survey 2001, 2006 and 2011.

## Methods

The Nepal Demographic and Health Survey (NDHS) is a nationally representative population based survey conducted every 5 years since 1986. The NDHS implemented two stage cluster sampling. Firstly, the country was divided into sample domains and secondly sampling units were selected in each sample domain with probability proportional to size. The household was selected randomly from the sampling unit. The overall response rates of these surveys were high: NDHS 2001 (97.8 %), NDHS 2006 (98.0 %) and NDHS 2011 (97.6 %). Details of the sampling procedure are mentioned in the individual survey reports [20–22].

Child datasets of the NDHS 2001, 2006 and 2011 were appended for analysis. The datasets contained information about under 5 year old children, their parent and household characteristics. A total of 12,845 last born children born within 5 years before individual surveys were included in this study irrespective of their current status (living or dead). Since the time of initiation of breastfeeding is applicable to only those children who were reported to be breastfed at least once, children who were never breastfed were excluded from analysis.

### Definitions of variables

WHO defines early initiation of breastfeeding as ‘provision of mother’s breast milk to infants within one hour of birth’ [23]. During the survey mothers were asked –*“How long after birth did you first put (name of the children) to the breast?”*The answers were recorded in terms of hours and days after birth. For this analysis, a child breastfed immediately after the birth and within first hour of birth was coded as “1 (Early initiation)” otherwise“0 (Late initiation)” [1, 4].

The main explanatory variable was mother’s education level which was recorded as: (i) no education (ii) primary education and (iii) secondary or higher education. Other independent variables were selected based on literature [19, 24]. Place of residence was reported as (i) urban and (ii) rural. Ecological zone was classified according to the altitude as (i) Mountain (ii) Hill (iii) Terai. Administrative regions are the five north-south segments of the nation namely (i) eastern development region (ii) central development region (iii) western development region (iv) mid-western development region and (v) far-western development region. Mother’s occupation was classified into 3 categories: (i) not working (ii) agriculture (iii) other paid jobs [24]. Father’s education was recorded similar to mother’s education. Religion was grouped into (i) Hindu and (ii) Others (Muslim, Buddhist, Christian). Ethnicity was firstly classified in to seven groups according to Nepal Demographic and Health Survey ethnicity classification [25] and was further grouped into four categories:(i) relatively advantaged (Brahmin/Chhetri) (ii) relatively disadvantaged (Janajati/Newar/Muslims) (iii) Disadvantaged (Dalits) and (iv) others (*Madheshi* and other unidentified) [25]. NDHS uses household assets to derive wealth index derived from principal component analysis [26] as a measure of socioeconomic status. This wealth score is then divided in to five quintiles (i) poorest (ii) poorer (iii) middle (iv) richer and (v) richest [22]. The mother’s age was grouped into three categories (i) < 20 year (ii) 25–34 years (iii) > =35 years. The sex of the child was grouped as: (i) male (ii) female. Birth order was recoded as: (i) first (ii) second (iii) third or more. Size at birth was based on the mother’s perception of the size of child at birth which was recorded as:(i) very small/small (ii) average or (iii) large/very large [13]. Variable multiple births is a dummy for twins/triplets. Place of delivery was classified as: (i) home (ii) health facility (government or private facilities) (iii) other places. Mode of delivery was recorded as: (i) caesarean (ii) vaginal.

The DHS are conducted in more than 50 countries, mostly in low and middle income countries, and serve as a major source of policy making in those contexts. A generic question was first prepared by measure DHSprogram and modified according to country specific context. In Nepal, the questionnaire was pre-tested and validated before the survey. More detail on the questionnaire can be found elsewhere [20–22, 27].

### Statistical analysis

**Fig. 1:**
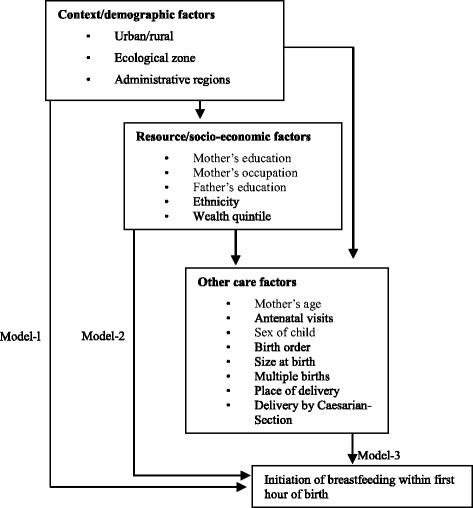
Conceptual framework of determinants of early initiation of breastfeeding, prepared for hierarchical logistic regression modeling. Based on the model developed by UNICEF and extended by Engle et al [28]. Adopted and from Matanda et al [29] with some modification

We adopted the conceptual framework from Matanda et al. [28] with some modifications (see Fig. 1) to facilitate our statistical analyses. This conceptual framework was originally based on the model developed by the United Nations Children’s Fund (UNICEF) and extended by Engle et al. [29]. The variables were grouped in to context, socio-economic and child specific demographic/health care variables according to their proximity to the dependent variable. We assumed that proximate variables have more direct effect than the distant variables. Statistical analysis was based on the survey analysis technique adjusting for weights assigned for non-response (sample weight) and cluster sampling design. Descriptive analysis was performed for sample background characteristics, followed by a logistic regression analysis to assess the association between mother’s education status and early initiation of breastfeeding, based on the conceptual framework. Context variables (urban/rural, ecological zone and administrative regions) were adjusted in model-1. In model-2; socioeconomic variables (mother’s occupation, fathers’ education status, religion, ethnicity, wealth quintile) and in model-3 child specific demographic and health care related variables (mother’s age, ANC utilization, sex of child, birth order of the child, the size of the child at birth, multiple birth, place of delivery, mode of delivery) were adjusted respectively. Variables found significant in the previous model (*P* < 0.05) were additionally adjusted in succeeding models until the final model (model-4) was obtained with all the significant variables by following the conceptual framework [30].

We checked the interaction term ‘survey year*mother’s education’ to assess the change in mother’s education status between the surveys, which was significant at *p* < 0.001. Therefore, independent analyses were also performed for each survey 2001, 2006 and 2011. *P*-value < 0.05 was considered statistically significant. Statistical analysis was performed in STATA 13.0.

### Ethics

NDHS obtained ethical approval from the Nepal Health Research Council, Kathmandu, Nepal and Macro Institutional Review Board, Maryland, USA [20–22]. NDHS obtained consent from mothers and their children before starting the interview. Permission to use the survey data for this study was granted by the Measure DHS program [27].

## Results

### Characteristics of respondents

**Fig. 2:**
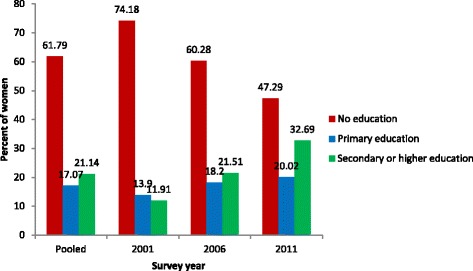
Percentage of ever breastfed women gave birth within 5 years before the survey according to their education status (NDHS 2001, 2006 and 2011)

The result represents a total of 12,845 mother-child pairs from demographic and health surveys. The majority of the respondents (58.68 %) had no education, about 17.50 % had primary and 23.82 % had a secondary or higher level education (see Fig. 2). Nearly nine in every ten respondents were from rural areas, more than half (51.01 %) were from Terai/plain land and about 31.93 % were from the central development region. About three quarters (75.33 %) of mothers were working in the agriculture sector. The majority of the children (61.29 %) were born from the mothers of age group 20–34 years. Only about 21.97 % children were delivered in a health facility (Table 1).

**Table 1:** Percentage of newborn babies breastfed within the first hour of birth according to the background characteristics. Children born within 5 years before the surveys NDHS 2001, 2006 and 2011

Characteristics	Total^a^	Pooled (*n* = 12,845)	NDHS 2011(*n* = 4116)	NDHS 2006(*n* = 4051)	NDHS 2001(*n* = 4678)	
	%	(n)	% early breastfed	% early breastfed	% early breastfed	% early breastfed
Survey year						
2001	36.42	4678	31.27			
2006	31.54	4051	35.26			
2011	32.05	4116	44.49			
Mother’s education						
No education	58.68	7537	31.55	34.42	31.54	30.04
Primary	17.5	2248	38.54	45.1	37.38	31.69
Secondary/higher	23.82	3060	48.29	56.33	42.73	37.57
Place of residence						
Urban	9.89	1270	41.44	48.42	39.44	35.89
Rural	90.11	11,575	36.25	44.05	34.63	30.92
Ecological zone						
Mountain	7.79	1001	37.62	47.82	31.26	35.01
Hill	41.2	5292	40.29	46.91	32.65	41.24
Terai	51.01	6552	33.78	42.16	38.08	22.44
Development region						
Eastern	22.97	2950	36	48.28	32.84	27.35
Central	31.93	4101	26.65	34.86	33.6	13.6
Western	19.29	2478	38.88	49.08	35.61	32.38
Mid-western	13.98	1796	48.62	45	32.38	63.85
Far-western	11.83	1520	48.08	54.73	44.7	46.23
Mother’s occupation						
Not working	20.84	2677	35.35	42.59	38.07	21.65
Agriculture	75.33	9676	36.88	44.61	34.15	33.25
Paid Jobs	3.83	492	42.29	58.85	41.87	30.91
Father’s education						
No education	26.74	3435	29.23	31.27	32.83	26.05
Primary	25.69	3300	34.99	41.1	33.01	31.81
Above secondary	46.41	5961	42.34	51.09	37.94	36.02
Don’t know	1.16	149	26.49	34.21	25.9	24.96
Ethnicity						
Advantaged	29.99	3852	43.24	51.18	33.21	44.92
Relatively disadvantaged	41.07	5275	38.71	45.32	40.87	30.57
Disadvantaged	15.52	1994	33.65	38.77	31.4	30.71
Madheshi and others	13.42	1724	19.95	29.85	27.03	9.41
Wealth quintile						
Poorest	20.91	2686	32.44	38.12	29.73	28.52
Poorer	20.55	2640	35.86	40.32	37.59	29.6
Middle	19.41	2493	37.57	43.05	34.68	34.71
Richer	19.63	2521	37.71	50.88	34.98	30.24
Richest	19.49	2503	40.61	54.47	41.1	32.76
Age of mother						
14–19 years	7.96	1022	36.06	45.26	39.54	24.65
20–34 years	61.29	7873	38.26	46.84	35.66	32.39
35–49 years	30.75	3950	33.97	39.03	33.16	30.9
ANC visit						
No Visits	31.71	4053	31.59	33.51	28.49	32.47
1–3 visits	37.71	4820	33.87	38.36	36.36	27.21
4 or more	30.59	3910	45.92	52.33	40.04	36.64
Sex of child						
Male	51.79	6652	37.03	44.98	35.39	31.11
Female	48.21	6193	36.48	43.94	35.12	31.42
Birth order of child						
First	26.01	3341	38.46	47.12	37.65	27.81
Second	25.8	3314	41.14	50.11	37.93	34.74
Third or more	48.19	6190	33.51	38.63	32.25	31.15
Multiple birth						
Single birth	98.8	12,691	36.96	44.82	35.53	31.31
Multiple births	1.2	154	20.95	21.63	18.14	25.31
Size at birth						
Very small/small	59.51	7644	35.84	43.96	33.79	29.23
Average	18.62	2392	35.84	42.67	31.64	34.63
Larger	21.81	2801	40.03	47.82	41.81	33.04
Place of delivery						
Home	76.35	9807	33.82	37.97	34.2	31.05
Health facility	21.97	2822	47.72	55.11	39.68	35.45
Others	1.67	215	27.37	31.02	33.6	21.44
Mode of delivery						
Vaginal	96.93	12,451	37.22	45.46	35.87	31.4
Caesarean	3.07	394	22.56	27.04	16.14	19.42

^a^numbers and percentages after adjusting for sample weight and cluster sampling design

### Percentage of children breastfed within the first hour after birth

**Fig. 3:**
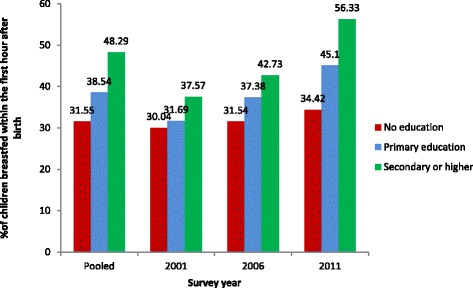
Percentage of last born children born within 5 years before the survey breastfed within the first hour after birth according to mother’s education status and survey year (NDHS 2001, 2006 and 2011)

The pooled data showed that slightly more than one third (36.77 %, 95 % CI: 35.79, 37.75) of the children were breastfed within the first hour after birth. Such an early initiation was higher for 2011 (44.49 %, 95 % CI: 42.65, 46.34) compared to 2006 (35.26 % 95 % CI: 33.45, 37.11) and 2001 (31.27 %, 95 % CI: 29.88, 32.69) (see Fig. 3). The difference in the percentage of women initiating breastfeeding within first after birth was statistically significant (*P* < 0.001) during the survey periods. The proportion of early initiation from pooled estimation was found to increase with increase in level of education. Results from the pooled data showed that children from the mothers with at least secondary education were more likely to be breastfed within 1 h after birth (48.29 %) in comparison to mothers with no education (31.55 %) and mothers with primary education (38.54 %) (see Fig. 3). A similar pattern was found, according to the father’s education status.

The percentage of newborn infants breastfed within the first hour after birth was higher among urban (41.44 %) areas in comparison to rural areas and the hill region (40.29 %) compared to the mountain or *Terai* region. Similarly, babies from the mothers with paid jobs, whose fathers had higher education, from the Hindu religion, from the advantaged ethnic group; from higher wealth quintiles had higher rates of early initiation of breastfeeding. The percentage of early breastfeeding was higher among the babies from mothers of age 20–34 years, mothers who completed four antenatal care visits, babies born in the second in order, babies average or larger in size at birth and babies delivered in a health facility. The proportion of early breastfed babies was lower among the multiple births and caesarean delivery compared to babies delivered by vaginal delivery. Detail comparison of proportions is presented in Table 1.

### Association between maternal education and early initiation of breastfeeding

Table 2 shows the results of bivariate and multivariate analysis adjusted for relevant variables. The table shows the results from the final model (model 4, according to conceptual framework) of logistic regression analysis for pooled data, data from NDHS 2011, NDHS 2006 and NDHS 2001. Pooled data provides us the higher power to detect the statistical association, if there is any. It also allows us to calculate narrow confidence intervals because of the larger sample size. The results of the logistic regression analysis using pooled data showed higher odds of early initiation of breastfeeding among children from the mothers with primary education [OR: 1.24; 95 % CI: 1.08, 1.42], and secondary or higher education [OR: 1.63; 95 % CI: 1.42, 1.88] compared to the children from mothers with no education (Table 2).

**Table 2:** Association between mother’s education status and initiation of breastfeeding within one hour after birth. Hierarchical modelling by using logistic regression analysis (pooled data, NDHS 2011, NDHS 2006 and NDHS 2001)

	Unadjusted from pooled analysis				Pooled Model^a^				2011^b^				2006^c^				2001^d^			
Characteristics	OR	(95 % CI)		*P*-value	OR	(95 % CI)		*P*-value	OR	(95 % CI)		*P*-value	OR	(95 % CI)		*P*-value	OR	(95 % CI)		*P*-value
Mother’s education																				
No education	1.00				1.00				1.00				1.00				1.00			
Primary	1.36	(1.20	1.54)	0.000	1.24	(1.09	1.42)	0.002	1.52	(1.21	1.91)	0.000	1.24	(0.96	1.60)	0.093	1.07	(0.87	1.32)	0.506
Above secondary	2.03	(1.79	2.30)	0.000	1.63	(1.42	1.88)	0.000	2.20	(1.76	2.76)	0.000	1.66	(1.30	2.12)	0.000	1.30	(1.00	1.70)	0.052
Place of residence																				
Urban	1.00																1.00			
Rural	0.80	(0.70	0.92)	0.002													0.74	(0.58	0.93)	0.012
Ecological zone																				
Mountain	1.00																1.00			
Hill	1.12	(0.96	1.30)	0.149													1.25	(1.02	1.53)	0.030
Terai	0.85	(0.71	1.01)	0.062													0.74	0.58	0.94)	0.013
Development region																				
Eastern	1.00				1.00				1.00				1.00				1.00			
Central	0.65	(0.55	0.76)	0.000	0.72	(0.62	0.83)	0.000	0.65	(0.47	0.91)	0.012	1.12	(0.90	1.40)	0.317	0.42	(0.34	0.53)	0.000
Western	1.13	(0.94	1.37)	0.202	1.11	(0.93	1.33)	0.262	1.01	(0.73	1.40)	0.958	1.14	(0.81	1.60)	0.456	1.14	(0.83	1.56)	0.428
Mid-western	1.68	(1.39	2.03)	0.000	1.98	(1.63	2.41)	0.000	1.03	(0.75	1.42)	0.852	1.24	(0.90	1.71)	0.188	4.42	(3.10	6.29)	0.000
Far-western	1.65	(1.27	2.13)	0.000	1.92	(1.49	2.48)	0.000	1.53	(1.09	2.15)	0.014	1.97	(1.25	3.10)	0.004	2.14	(1.64	2.79)	0.000
Ethnicity																				
Advantaged	1.00				1.00								1.00				1.00			
Relatively disadvantaged	0.83	(0.72	0.96)	0.011	1.21	(1.04	1.41)	0.013					1.71	(1.29	2.26)	0.000	1.12	(0.87	1.45)	0.366
Disadvantaged	0.67	(0.57	0.78)	0.000	0.94	(0.80	1.10)	0.435					1.20	(0.89	1.61)	0.224	0.86	(0.69	1.07)	0.168
Madheshi and others	0.33	(0.26	0.42)	0.000	0.60	(0.47	0.76)	0.000					0.99	(0.68	1.44)	0.963	0.49	(0.28	0.83)	0.009
Wealth quintile																				
Poorest	1.00				1.00								1.00				1.00			
Poorer	1.16	(1.01	1.34)	0.036	1.21	(1.04	1.41)	0.015					1.42	(1.09	1.86)	0.010	0.96	(0.77	1.21)	0.746
Middle	1.25	(1.07	1.47)	0.006	1.27	(1.08	1.50)	0.003					1.18	(0.90	1.57)	0.231	1.11	(0.86	1.43)	0.432
Richer	1.26	(1.06	1.50)	0.008	1.24	(1.04	1.48)	0.019					1.21	(0.86	1.70)	0.265	0.95	(0.73	1.25)	0.730
Richest	1.42	(1.21	1.68)	0.000	1.24	(1.04	1.47)	0.017					1.45	(1.06	1.99)	0.022	1.03	(0.78	1.35)	0.845
ANC visit																				
No visits	1.00				1.00								1.00				1.00			
1–3 visits	1.11	(0.97	1.26)	0.122	1.07	(0.94	1.21)	0.302					1.36	(1.10	1.67)	0.004	0.95	(0.82	1.11)	0.530
4 or more	1.84	(1.58	2.14)	0.000	1.32	(1.12	1.56)	0.001					1.40	(1.04	1.89)	0.026	1.38	(1.07	1.78)	0.014
Birth order																				
First	1.00				1.00				1.00								1.00			
Second	1.12	(0.99	1.26)	0.061	1.30	(1.15	1.47)	0.000	1.43	(1.15	1.78)	0.002					1.53	(1.23	1.91)	0.000
Third or more	0.81	(0.72	0.90)	0.000	1.22	(1.08	1.38)	0.001	1.29	(1.02	1.63)	0.036					1.35	(1.14	1.61)	0.001
Size at birth																				
Small	1.00												1.00							
Average	1.00	(0.89	1.12)	0.998									0.97	(0.77	1.21)	0.757				
Larger	1.19	(1.03	1.38)	0.017									1.33	(1.03	1.71)	0.027				
Multiple births	0.45	(0.25	0.81)	0.007	0.42	(0.23	0.76)	0.004	0.26	(0.08	0.81)	0.021	0.38	(0.13	1.11)	0.078				
Place of delivery																				
Home	1.00				1.00				1.00								1.00			
Health facility	1.79	(1.58	2.02)	0.000	1.73	(1.50	2.01)	0.000	2.09	(1.74	2.50)	0.000					1.39	(1.06	1.82)	0.018
Other place	0.74	(0.54	1.02)	0.062	0.67	(0.47	0.94)	0.022	0.62	(0.33	1.16)	0.133					0.42	(0.23	0.77)	0.005
Mode of delivery																				
Vaginal	1.00				1.00				1.00				1.00				1.00			
Caesarian	0.49	(0.36	0.67)	0.000	0.27	(0.19	0.38)	0.000	0.24	(0.16	0.36)	0.000	0.24	(0.12	0.48)	0.000	0.40	(0.16	0.99)	0.047

*OR* odds ratio; *CI* confidence interval

^a^ Adjusted for- Mother’s education, Development region, Ethnicity, Wealth quintile, ANC visit, Birth order, Multiple births, Place of delivery and Mode of delivery

^b^ Adjusted for-Mother’s education, Development region, Birth order, Multiple births, Place of delivery and Mode of delivery

^c^ adjusted for -Mother’s education, Development region, Ethnicity, Wealth quintile, ANC visits, Size at birth, Multiple births, Mode of delivery

^d^ adjusted for-Mother’s education, Place of residence, Ecological zone, Development region, Ethnicity, Wealth quintile, ANC visit, Birth order, Place of delivery and Mode of delivery

We assessed the interaction term between the mother’s education and survey year, in the multivariate adjusted model, since the interaction was significant (*p* < 0.001) we developed separate models for individual survey 2011; 2006, and 2001. In 2011, odds of early initiation of breastfeeding was higher among mothers with primary education [OR: 1.52; 95 % CI: 1.21, 1.91] and secondary or higher education [OR: 2.20; 95 % CI: 1.76, 2.76] compared to those with no education. In 2006, it was also higher among mothers with secondary or higher education [OR: 1.66; 95 % CI: 1.30, 2.12]. In 2001, having a secondary or higher education was marginally significant [OR = 1.30; 95 % CI 1.00, 1.67; *p* = 0.05]. These models showed that there was increased likelihood of early initiation of breastfeeding with increasing maternal education.

This analysis found other characteristics independently associated with early initiation of breastfeeding, after controlling for the effect of mother’s education. The groups who had a higher likelihood of early initiation were the mothers who were from mid-western and far-western regions, from higher wealth quintile, who had four antenatal care visits, were second parity mothers, had larger babies, and had delivered in health facilities. Conversely, mothers who were from the Terai (plain) region, rural areas, from madheshi and other ethnic group, had multiple births and had delivered via caesarean section had a lower likelihood of early initiation of breastfeeding. (Details are shown in the Additional file 1).

## Discussion

This study found newborn babies from the mothers with primary education and secondary or higher education were more likely to be breastfed within the first hour after birth in comparison to the babies from the mothers with no education. We found a significant increase in the percentage of early initiation of breastfeeding during three surveys. National nutrition policy and strategy 2004 and its amendment in 2008 have given special emphasis on breastfeeding promotion including early initiation of breastfeeding [31], also mentioned in the Multi-Sectoral Nutrition Plan (2013–2017) [32]. Community based newborn care program (CB-NCP) implemented by the government directly advocates for the initiation of breastfeeding within first hour after birth [33, 34]. The government has promoted Baby Friendly Hospital Initiative [35–37]. Public awareness campaigns via mass media might also have impacted to this increase in early initiation of breastfeeding. A recent study conducted in central Nepal reported that about 67 % of recently delivered mothers breastfed their newborns within the first hour of birth [12]. A cross sectional survey conducted in an urban city of western Nepal in 2006 found that about 58 % mothers initiated breastfeeding their babies within first hour after birth [38]. These studies reported higher rates of early initiation than the NDHS and the difference can be accounted for in varied study settings and methodology. This study confirms the positive role of mother’s education to early initiation of breastfeeding. The association between mother’s education and early initiation of breastfeeding was at a marginal significance level for mothers with secondary or higher education in 2001 and was statistically significant in 2006. Meanwhile, the association was more prominent for the latest 2011 survey. This shows the increasing importance of education for uptake of ‘messages’ about breastfeeding that emerged in government policy and various national programmes in between the survey periods. An observational study from Bangladesh reported that caretakers with formal education were more likely to feed their babies more frequently, feed hygienic foods in a hygienic environment [39]. A review of literature had reported maternal education as a consistently associated demographic factor with early initiation of breastfeeding [19]. A systematic review of interventions designed to promote exclusive breastfeeding in high income countries found that the interventions using educational approaches were significantly associated to the increase in the duration of exclusive breastfeeding [40, 41]. Educating and counselling mothers have found as a successful intervention for early initiation and sustaining breastfeeding even in the complicated situation like caesarean section [42]. Taken together, the current findings of a consistent association of maternal education with early initiation of breastfeeding is comparable to international findings.

### Other significant factors associated with early initiation of breastfeeding

It is worthwhile to discuss the other demographic and socio-economic characteristics identified as independently associated with the early initiation of breastfeeding. However, the primary objective of this paper was to investigate the association between mother’s education and early initiation of breastfeeding.

Higher odds of early initiation of breastfeeding in the western and far western region can be explained by the concentrated effort of existing maternal and child health intervention programs in the recent years. It might also because of the unavailability or inaccessibility to the alternatives of breastfeeding in those regions.

The odds of early initiation were higher among higher wealth quintiles. Socio- economic deprivation has been identified as the predictor of breastfeeding duration elsewhere [43, 44]. Higher wealth index was found positively associated with the greater likelihood of using recommended level of antenatal care visits [45], where they receive counselling about colostrum feeding. Wealthier mothers are also more likely to deliver at the health facility and receive postnatal care where they can get more support for breastfeeding initiation [46, 47], while poorest mothers are deprived from those advantages.

Higher odds of early initiation of breastfeeding among the babies delivered at health facilities can be explained by the presence of health professional, their monitoring and support for the breastfeeding, which assures mothers to start early initiation of breastfeeding. This study found a significant proportion of children delivered at health facility were still not breastfed within first hour after birth. A study reported found higher rate of early initiation of breastfeeding and better newborn care practices among the institutional delivery in Dhanusha district (from the Terai region) of Nepal [48]. However, the study also showed the scope for improvement on the early initiation of breastfeeding among institutional deliveries [48].

Similarly, large size babies are more likely to be full termed and healthier, they have better coordination of the suction-deglutition-respiration cycle and breast seeking reflex [49, 50]. This explains why the large size babies are more likely to be early breastfed than small size babies.

Support from the family and society is important factor for successful breastfeeding. Women’s knowledge about the benefit of breastfeeding and their perception plays an important role to initiate breastfeeding early. Women in rural areas are found to have low knowledge about the importance of breastfeeding and they are also less likely to get support for breastfeeding. Those factors might have resulted in lower odds of early initiation of breastfeeding in the rural areas [51]. Similarly, lower odds of early initiation of breastfeeding in the terai region may be associated with the cultural practices of introduction of prelacteal food in the region [13]. An earlier study from Rupandehi district of Nepal (from the terai region) also reported extremely low rate (3.4 %) of initiation of breastfeeding within the first hour after birth [9].

A study in southern Nepal, where most of the participants were from Madheshi ethnic group revealed that about two in five mothers discarded colostrum [52]. Such harmful cultural practices inhibit initiation of breastfeeding within first hour after birth; therefore we obtained lower odds of early initiation of breastfeeding among those ethnic groups.

It may be difficult for mothers to breastfeed early who are primi-parous, have multiple births and have a caesarean delivery to initiate breastfeeding within the first hour of birth. This may explain the lower odds of early initiation among caesarean deliveries [53–56]. Earlier studies have found parity associated with the breastfeeding behaviour [57, 58]. Confidence of mother is also an important factors which determines the initiation of breastfeeding, which increases with the parity [56].

### Public health implications of the current findings

The current findings have a number of public health implications. This study has demonstrated the importance of maternal education on early initiation of breastfeeding. Since, improving educational status of mothers is a long term agenda, immediate attention should be provided to mothers with no or less education with alternative supportive and educational interventions like prenatal education and counselling [59]. Midwives, other health professionals and nurses can play important role to encourage mothers for early initiation of breastfeeding [60].

This study is based on the three large, nationally representative population based Demographic and Health Surveys in Nepal. Large sample size ensures a high precision of the findings. NDHS used the standardized tools; therefore, the results are reliable and comparable with other developing countries. However, causal inference between maternal education level and early initiation of breastfeeding is limited due to the study being cross sectional. Similarly, maternal education here represents the formal education gained through schooling and it may not reflect the health literacy of the mothers. This study doesn’t explain about the separate counselling or breastfeeding education provide to women during the antenatal or postnatal period, which might have impacted on the early initiation of breastfeeding. Counselling and breastfeeding education are the major components of antenatal care visit (ANC), which is however, included in this study. Also, there may be recall bias since this study is based on the interview with mothers who gave birth within 5 years before the survey.

## Conclusions

Maternal education was associated with an early initiation of breastfeeding in the 2001, 2006 and 2011 studies. Mothers with higher education were more likely to initiate breastfeeding with the first hour of childbirth. Future interventions should focus on increasing girl’s and women’s education program through formal or non-formal education programs, respectively. While school education is feasible for a girl child, adult learning approaches using literacy programs for adults, counselling, peer educations using through peer education would be more suitable for adult women who did not have a chance to go to school during their childhood.

## Additional file

Additional file 1:
**Detail results of the hierarchical modeling using logistic regression.** (XLSX 35 kb)

## Abbreviations

ANC: Antenatal Care
CI: Confidence interval
NDHS: Nepal Demographic and Health Survey
OR: Odds ratio
WHO: World Health Organization
